# Synthesis and evaluation of 2-chloro *N*-[(*S*)-{(*S*)-1-[^11^ C]methylpiperidin-2-yl} (phenyl)methyl]3-trifluoromethyl-benzamide ([^11^ C]*N*-methyl-SSR504734) as a PET radioligand for glycine transporter 1

**DOI:** 10.1186/2191-219X-2-37

**Published:** 2012-07-09

**Authors:** Takeshi Fuchigami, Akihiro Takano, Balázs Gulyás, Zhisheng Jia, Sjoerd J Finnema, Jan D Andersson, Ryuji Nakao, Yasuhiro Magata, Mamoru Haratake, Morio Nakayama, Christer Halldin

**Affiliations:** 1 Graduate School of Biomedical Sciences, Nagasaki University, Nagasaki, 852-8521, Japan; 2 School of Medicine, Medical Photonics Research Center, Hamamatsu University, Hamamatsu, 431-3192, Japan; 3 Karolinska Institutet, Department of Clinical Neuroscience, Center for Psychiatric Research, Karolinska University Hospital, R5:02, Stockholm 171 76, Sweden

**Keywords:** Glycine transporter 1, SSR504734, ^11^ C, Positron emission tomography, Schizophrenia

## Abstract

**Background:**

Dysfunction of the glycine transporter 1 (GlyT1) has been suggested to be involved in psychiatric disorders such as schizophrenia. GlyT1 inhibitors have therefore been considered to have antipsychotic therapeutic potential. Positron emission tomography (PET) imaging probes for GlyT1 are, consequently, expected to be useful for investigating the mechanism of such disease conditions and for measuring occupancy of GlyT1 inhibitors *in vivo*. The aim of this study was to assess the potential of 2-chloro *N*-[(*S*)-{(*S*)-1-[^11^ C]methylpiperidin-2-yl} (phenyl)methyl] 3-trifluoromethyl-benzamide ([^11^ C]*N*-methyl-SSR504734) as a PET imaging agent for GlyT1.

**Methods:**

[^11^ C]*N*-methyl-SSR504734 was synthesized by *N*-[^11^ C]methylation of SSR504734 via [^11^ C]CH_3_OTf. *In vitro* brain distribution of [^11^ C]*N*-methyl-SSR504734 was tested in whole-hemisphere autoradiography (ARG) on human brain slices. Initial PET studies were performed using a cynomolgus monkey at baseline and after pretreatment with 0.1 to 1.5 mg/kg of SSR504734. Then, PET studies using rhesus monkeys were performed with arterial blood sampling at baseline and after pretreatment with 1.5 to 4.5 mg/kg SSR504734. Distribution volumes (*V*_T_) were calculated with a two-tissue compartment model, and GlyT1 occupancy by SSR504734 was estimated using a Lassen plot approach.

**Results:**

[^11^ C]*N*-methyl-SSR504734 was successfully synthesized in moderate radiochemical yield and high specific radioactivity. In the ARG experiments, [^11^ C]*N*-methyl-SSR504734 showed specific binding in the white matter and pons. In the initial PET experiments in a cynomolgus monkey, [^11^ C]*N*-methyl-SSR504734 showed high brain uptake and consistent distribution with previously reported GlyT1 expression *in vivo* (thalamus, brainstem > cerebellum > cortical regions). However, the brain uptake increased after pretreatment with SSR504734. Further PET studies in rhesus monkeys showed a similar increase of brain uptake after pretreatment with SSR504734. However, the *V*_T_ of [^11^ C]*N*-methyl-SSR504734 was found to decrease after pretreatment of SSR504734 in a dose-dependent manner. GlyT1 occupancy was calculated to be 45% and 73% at 1.5 and 4.5 mg/kg of SSR504734, respectively.

**Conclusions:**

[^11^ C]*N*-methyl-SSR504734 is demonstrated to be a promising PET radioligand for GlyT1 in nonhuman primates. The present results warrant further PET studies in human subjects.

## Background

Glycine is a neurotransmitter of the inhibitory glycine receptors (GlyRs) but also serves as an essential co-agonist of the excitatory* N*-methyl-_D_-aspartate (NMDA) receptors [1,2]. It is known that the extracellular glycine concentration is modulated by two glycine transporters (GlyT1 and GlyT2) [3]. It has been suggested that GlyT2 is mainly implicated in the control of inhibitory neurotransmission via GlyRs, whereas GlyT1 is considered to regulate the glycine level in both inhibitory neuron and excitatory neurons [4-8].

**Figure 1:**
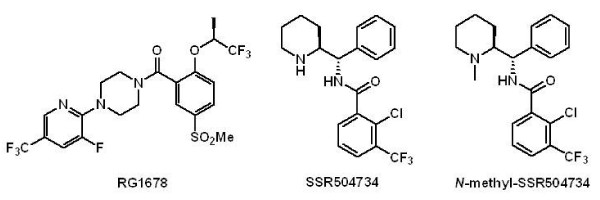
Chemical structure of GlyT1 inhibitors.

It has been indicated that NMDA receptor dysfunction may be related to various disorders such as schizophrenia, Parkinson's Disease, and Alzheimer's Disease [9-11]. Schizophrenia has been hypothesized to be related to the hypofunction of NMDA receptors. Direct NMDA receptor agonists would cause severe side effects. Targeting the glycine site of the NMDA receptors has been suggested to be a promising therapeutic strategy through the facilitation of NMDA receptor functions [12-14]. Indeed, it was reported that enhancement of glycine concentration in the synaptic cleft by GlyT1 inhibitors, such as RG1678 (Figure 1), was efficacious in the treatment of the negative and cognitive symptoms of schizophrenia [11,15,16]. In addition, GlyT1 inhibitors could be potential therapeutic agents for various disorders associated with inhibitory neurotransmission (e.g., depression and neuropathic pain) [17,18].

**Figure 2:**
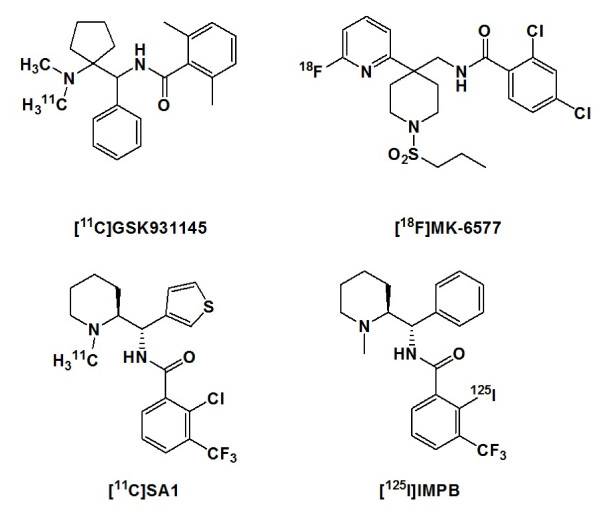
Chemical structure of PET radioligands for GlyT1.

Positron emission tomography (PET) is considered as one of the most useful imaging methods for noninvasive visualization of target proteins. Development of PET radioligands for GlyT1 is thus considered useful for obtaining information about various psychiatric diseases and occupancy of GlyT1 inhibitors *in vivo*. Several novel PET radioligands with high affinity to GlyT1 were reported recently. Among these radioligands, ^11^ C]GSK931145 and ^18^ F]MK-6577 (Figure 2) showed good brain uptake, good accumulation in GlyT1-rich regions, and significant blocking by GlyT1 ligands in the brain of pigs, rhesus monkeys, and humans [19-21].

The *N*-[Phenyl (piperidin-2-yl) methyl]benzamide derivative SSR504734 (Figure 1) is a selective and competitive inhibitor of GlyT-1 [22]. Since SSR504734 has moderate lipophilicity and a molecular weight of 397, SSR504734 may be a useful lead compound for the development of PET radioligands. Indeed, SSR504734-related radioligands such as ^11^ C]SA1 showed high brain uptake and a consistent accumulation with the GlyT1 distribution in the mouse and monkey brain [23]. More recently, we developed ^125^I]2-iodo *N*-[(*S*)-{(*S*)-1-methylpiperidin-2-yl} (phenyl)methyl]3-trifluoromethyl-benzamide (^125^I]IMPB) as a promising SPECT ligand candidate [24].

It was previously reported that *N*-methyl-SSR504734 has high affinity for GlyT1 (1.6 nM) [25] and a potent GlyT1 inhibitory activity (IC_50_ = 2.4 nM) [24]. The same structure with ^11^ C]*N*-methyl-SSR504734 was reported recently [23]. *In vivo*^11^ C]*N*-methyl-SSR504734 showed high brain uptake; however, PET evaluation using nonhuman primates were not so far reported in the literature [23]. In this study, we aimed to synthesize ^11^ C]*N*-methyl-SSR504734 and evaluate its distribution by *in vitro* autoradiography in human brain slices and by *in vivo* PET studies in monkeys.

## Methods

### General

SSR504734 and *N*-methyl-SSR504734 were synthesized according to the literature [25,26]. All other chemicals were obtained from commercial sources, were of analytical grade, and were used as received. Purification of ^11^ C]*N*-methyl-SSR504734 was performed by a high-performance liquid chromatography (HPLC) system consisting of a Merck-Hitachi absorbance detector (*λ* = 254 nm; VWR International, Stockholm, Sweden) in series with a GM tube (Carroll-Ramsey, Berkely, CA) for radioactivity detection. The purified ^11^ C]*N*-methyl-SSR504734 was analyzed by a HPLC system consisting of a Merck-Hitachi L-7100 Pump, L-7400 UV detector, and GM tube for radioactivity detection (VWR international). The radiometabolism of each product in monkey plasma was assessed with HPLC on a system that contained a Merck-Hitachi D-7000 interface module, L-7100 pump and an L-7400 absorbance detector (*λ* = 254 nm; Merck-Hitachi, Tokyo, Japan), and an injector (7125 with a 5.0-ml loop; Rheodyne, Cotati, CA, USA) equipped with a μ-Bondapack-C18 column (300 × 7.8 mm, 10 μm; Waters, Milford, MA, USA) in series with a 150TR radiodetector (Packard Radiomatic, PerkinElmer, Waltham, MA, USA) equipped with a PET flow cell (550 μl).

### Preparation of [^11^ C]*N*-methyl-SSR504734

Ultrahigh specific activity of ^11^ C]CH_3_I and ^11^ C]CH_3_OTf was prepared from ^11^ C]CH_4_ as described previously [27]. In brief, ^11^ C]CH_4_ was obtained via ^14^ N(p,a)^11^ C reaction on nitrogen containing 10% hydrogen with 16 MeV protons. ^11^ C]CH_4_ reacted with iodine at high temperature in a heated quartz column and converted to ^11^ C]CH_3_I. ^11^ C]CH_3_OTf was prepared by carrying ^11^ C]CH_3_I vapor through a heated glass column containing silver triflate-impregnated graphitized carbon. The ^11^ C]CH_3_I or ^11^ C]CH_3_OTf was trapped in a vessel containing SSR504734 (0.6 mg, 1.51 μmol), organic solvent (DMF, acetone or CH_3_CN; 400 μl), and base (NaH or NaOH; 5 μmol). After 3 min, the radioactive reaction mixture was purified by an HPLC (column, ACE5 C18-HL, 250 × 10 mm; mobile phase, CH_3_CN: 0.1 M NH_4_HCO_2_ in H_2_O = 45:55; flow rate, 6.0 ml/min). The solvents in the collected product fraction were evaporated at 70°C, and the final product was formulated in phosphate buffered saline (PBS; pH = 7.4). The purity of the product was analyzed by an HPLC (column, μ-Bondapak C18 10 μm 125 Å 7.8 × 300 mm; mobile phase, CH_3_CN: 0.1% TFA in H_2_O = 30:70; flow rate, 2.0 ml/min) and identified by co-injection of reference *N*-methyl-SSR504734.

### *In vitro* autoradiography

Human brains without pathology were obtained from the National Institute of Forensic Medicine, Karolinska Institutet (Stockholm, Sweden) as well as from the Department of Forensic and Insurance Medicine, Semmelweis University, Budapest. After the removal of the brains (6- and 11-h post mortem times), they were kept at −85°C until sectioning; after which, the whole hemisphere brain slices were kept at −25°C until the autoradiographic procedures. Ethical permissions were obtained from the relevant research ethics committee of the respective institutions. The sectioning of the brains and the autoradiography experiments were performed at the Department of Clinical Neuroscience, Karolinska Institutet. The cryosectioning took place on a Leica cryomacrocut system (San Marcos, CA, USA) [28,29]. Horizontal sections (100 μm thick, containing cerebral cortex, white matter, cerebellum, and pons) and coronal sections (100 μm thick, containing cerebral cortex, white matter, putamen, and caudate nucleus) were used. The sections were incubated in binding buffer (120 mM NaCl, 2 mM KCl, 1 mM MgCl_2_, 1 mM CaCl_2_, and 50 mM Tris–HCl, pH 7.5) containing ^11^ C]*N*-methyl-SSR504734 (40 MBq) at 25 °C for 30 min. To determine the nonspecific binding, unlabelled *N*-methyl-SSR504734 (10 μM) was included in the incubation buffer for anatomically adjacent sections. For comparison with other GlyT1 radioligands, blocking was also made with SSR504734 (10 μM). The slices were rinsed thrice for 3 min each with cold (5°C) washing buffer (120 mM NaCl, 50 mM Tris–HCl, pH = 7.5) and subsequently dipped into cold water. The slides were then placed on an imaging plate (Fujifilm Plate BAS-TR2025, Fujifilm, Tokyo, Japan) for 20 min. The plates were developed, and the resulting images were processed in a Fujifilm BAS-5000 phosphorimager (Fujifilm, Tokyo, Japan). Aliquots (20 μl) of the incubation solution were spotted onto polyethylene-backed absorbent paper (BenchGuard™, Lennox, Dublin, Ireland), allowed to dry, scanned, and digitized in the phosphorimager parallel with the tissue scans. The autoradiography (ARG) signal's optical density was measured using Multi Gauge 3.2 image analysis software (Fujifilm, Tokyo, Japan).

### PET studies in monkeys

PET measurements were performed using a high-resolution research tomograph (CTI/Siemens Molecular Imaging, Knoxville, TN, USA) with an in-plane resolution less than 1.5 mm FWHM in the center of the FOV [30]. PET data were acquired by list mode. The data were reconstructed with a series of frames (5 × 1 min, 5 × 3 min, 5 × 6 min, and 13 × 10 min) using the Ordinary Poisson-3D-Ordered Subset Expectation Maximization (OP03D-OSEM) algorithm with 10 iterations and 16 subsets including modeling of the points spread function. Image reconstruction was performed on a quad-core PC running a fast reconstruction algorithm [31].

One female cynomogus monkey (4,900 and 5,200 g at different PET days) and two female rhesus monkeys (5,550 and 5,550 g) were used in this study. The study was approved by the Animal Ethics Committee of the Swedish Animal Welfare Agency (Dnr 145/08 and 386/09) and was performed according to the ‘Guidelines for Planning, Conducting and Documenting Experimental Research’ (Dnr 4820/06-600) of the Karolinska Institutet as well as the ‘1996 Guide for the Care and Use of Laboratory Animals’.

Anesthesia was induced by intramuscular injection of ketamine hydrochloride (60 mg) and maintained by the administration of a mixture of sevoflurane, O_2_, and medical air after endotracheal intubation. The head was immobilized with a fixation device. Body temperature was maintained by Bair Hugger Model 505 (Arizant Healthcare, MN, USA) and monitored by an esophageal thermometer. ECG, heart rate, respiratory rate, and oxygen saturation were continuously monitored throughout the experiments. Blood pressure was monitored every 5 min.

Magnetic resonance images (MRIs) of each monkey brain were previously obtained using a 1.5 T General Electrics Signa system (GE, Milwaukee, WI, USA). PET measurements consisted of two parts, as follows.

#### PET measurements using a cynomolgus monkey

PET measurements were performed for 123 min immediately after intravenous (i.v.) injection of radioligand at baseline and after pretreatment with SSR504734. SSR504734 was administered over 15 min, starting 30 min before the radioligand injection. PET measurements were performed on two separate days at a 6-week interval. One baseline PET measurement and one PET measurement after pretreatment with 0.1 mg/kg of SSR504734 were performed on the first day. One baseline PET measurement and two PET measurements after pretreatment with 0.5 and 1.5 mg/kg of SSR504734 were performed on the second day.

During each PET measurement, venous blood samples were taken from a femoral vein for the measurement of [^11^ C] *N*-methyl-SSR504734 protein binding at 5 min before the radioligand injection and for metabolite analysis of [^11^ C]*N*-methyl-SSR504734 at 4,15, 30, 60, 90, and 120 min after the radioligand injection.

#### PET measurements using rhesus monkeys

PET measurements were performed for 123 min immediately after i.v. injection of radioligand at baseline and after pretreatment with SSR504734. SSR504734 was administered over 15 min, starting 30 min before the radioligand injection. PET measurements were performed on two separate days with two monkeys. One baseline PET measurement and one PET measurement after pretreatment with 1.5 mg/kg of SSR504734 were performed on one monkey. One baseline PET measurement and one PET measurement after pretreatment with 4.5 mg/kg were performed on the other monkey.

During each PET measurement, arterial blood was collected continuously for 3 min using an automated blood-sampling system at a speed of 3.0 mL/min (Allog AB, Molndal, Sweden). Blood samples (1.0 to 2.5 mL) were drawn at 4, 15, 30, 60, 90, and 120 min after the radioligand injection for blood and plasma radioactivity and metabolite correction. One arterial blood sample (2.0 ml) was taken 5 min before the radioligand injection for the measurement of [^11^ C]*N*-methyl-SSR504734 protein binding.

### Metabolite analysis

From the obtained blood samples, plasma was obtained after centrifugation at 2,000 × *g* for 2 to 4 min and was mixed with 1.4 times the volume of acetonitrile. The supernatant acetonitrile-plasma mixture obtained after centrifugation at 2,000 × *g* for 2 to 4 min was injected into the radio-HPLC system. HPLC analysis was performed on a Waters μ-Bondapak-C_18_ column (300 × 7.8 mm, 10 μm) by gradient elution using (a) ammonium formate (100 mM) and (b) acetonitrile at 6.0 mL/min.

### Measurement of protein binding

Monkey plasma (500 μL), or PBS (500 μL) as a control, was mixed with [^11^ C]*N*-methyl-SSR504734 solution (50 μL, approximately 1 MBq) and incubated at room temperature for 10 min. Samples (20 μL) from each incubation mixture were measured with a well counter. After the incubation, 200-μL portions of the incubation mixtures were pipetted into ultrafiltration tubes (Millipore Centrifree YM-30, Merck Millipore, Billerica, MA, USA) and centrifuged for 15 min at 1,500 × *g*. Samples (20 μL) from each filtrate were pipetted for counting. The results were corrected for the membrane binding as measured with the control samples.

### Data analysis

Regions of interest (ROI) for the thalamus, cerebellum, caudate, putamen, frontal cortex, temporal cortex, anterior cingulate cortex, pons, white matter, and the whole-brain contour were delineated on PET/MRI co-registered brain images for each monkey. Co-registrations and ROI delineations were performed using PMOD 3.0 (Pixel-Wise Modeling Computer Software, PMOD Group, Zurich, Switzerland). PET data were analyzed using several parameters such as percent injected dose (%ID) and percent standard uptake value (%SUV), where

$$\begin{array}{ccc} \;\%\text{ID} & \;= & \frac{\text{total radioactivity in the whole brain (MBq})}{\text{injected radioactivity (MBq})}\\ & \;\times & 100 \end{array}$$

$$\begin{array}{ccc} \;\%\text{SUV} & \;= & \frac{\text{radioactivity (MBq/cc})}{\text{injected radioactivity (MBq})}\\ & \;\times & \text{body weight }\left(\text{g}\right)\times 100. \end{array}$$

In the PET measurements using rhesus monkeys, the total distribution volume (*V*_T_) was calculated by a two-tissue compartment model as well.

GlyT1 occupancy was estimated by applying Lassen plot approach [32], which can determine nondisplaceable distribution volume (*V*_ND_) and occupancy using *V*_T_ values at baseline and after pretreatment. Seven regions (the pons, cerebellum, putamen, frontal cortex, caudate, anterior cingulate cortex, and temporal cortex) were used for the plot.

## Results

### Radiochemistry

**Scheme 1:**
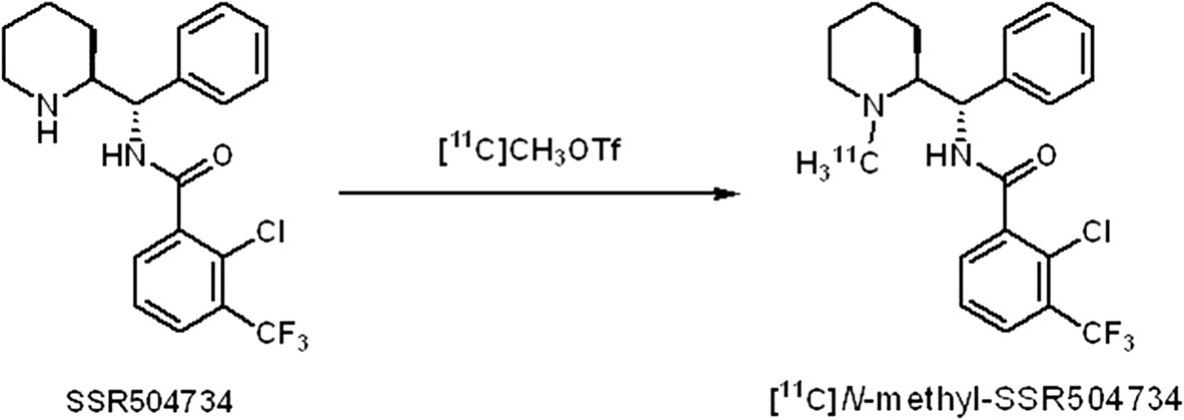
**Radiosynthesis of [**^11^ **C] *N* **-**methyl-SSR504734.**

When ^11^ C]CH_3_I was applied as a methylating agent and NaH or NaOH as a base, the desired ^11^ C]*N*-methyl-SSR504734 was not obtained in significant yield. On the other hand, in the case where ^11^ C]CH_3_OTf was used in the reaction instead of ^11^ C]CH_3_I, ^11^ C]*N*-methyl-SSR504734 was successfully synthesized. The highest radiochemical yield (36% to 42%, decay corrected from ^11^ C]CH_3_OTf) was achieved by using NaOH as a base and CH_3_CN as a solvent (Table 1 and Scheme 1). Specific activity of the ligand was calculated to be 1,420 to 1,860 GBq/μmol (*N* = 3) at the time when the synthesis of ^11^ C]*N*-methyl-SSR504734 was completed. The ultrahigh specific activity of the radioligand was consistent with our previous results by using ^11^ C]CH_4_ as a starting precursor [27].

**Table 1:** **Synthetic condition for preparation of [**^**11**^ **C] *N* -methyl-SSR504734**

**Entry**	**[**^**11**^ **C] methylating agent**	**Base**	**Solvent**	**Temperature**	**Time (min)**	**Yield (%)**
1	[^11^ C]CH_3_I	NaH	DMF	r.t.	5	0
2	[^11^ C]CH_3_I	NaOH	DMF	r.t.	5	0
3	[^11^ C]CH_3_I	NaOH	DMF	80 °C	5	0
4	[^11^ C]CH_3_OTf	NaOH	Acetone	r.t.	1	25
5	[^11^ C]CH_3_OTf	NaOH	CH_3_CN	r.t.	1	36 to approximately 42

*DMF*, dimethylformamide; r.t., room temperature.

### *In vitro* ARG of human brain slices

**Figure 3:**
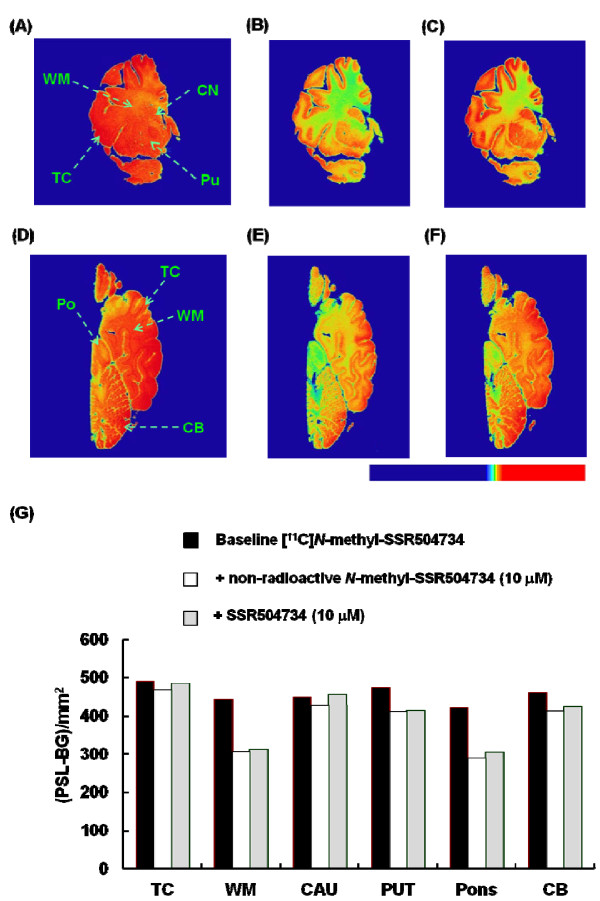
** *In vitro* postmortem autoradiography study of human brain. **Autoradiogram from coronal (panels **A**, **B**, and **C**; 100-mm thickness) and horizontal whole-hemisphere (panels **D**, **E**, and **F**; 100-mm thickness) cryosections of the human brain incubated with [^11^ C]*N*-methyl-SSR504734 under baseline (panels **A** and **D**), blocked by non-radioactive *N*-methyl-SSR504734 (10 μM; panels **B** and **E**), and blocked by SSR504734 (10 μM; panels **C** and **F**) conditions. The quantified values of [^11^ C]*N*-methyl-SSR504734 (**C**) were expressed as (PSL-BG)/mm^2^ (panel **G**). TC, temporal cortex; PUT, putamen; CAU, caudate nucleus; CB, cerebellum; WM, white matter.

In the postmortem autoradiography study *in vitro*, [^11^ C]*N*-methyl-SSR504734 showed a rather homogenous accumulation in the human brain slices including the temporal cortex, white matter, caudate nucleus, putamen, pons, and cerebellum (Figure 3A,D). The reduction of [^11^ C]*N*-methyl-SSR504734 binding by non-radioactive *N*-methyl-SSR504734 and SSR504734 was prominent in the white matter and pons, but in other regions, the degree of the reduction was small (Figure 3).

### PET measurements using a cynomolgus monkey

The injected radioactivity was 144.4 ± 16.5 MBq. Specific radioactivity was higher than 234 GBq/μmol at the time of injection. The corresponding injected mass was less than 0.3 μg.

**Figure 4:**
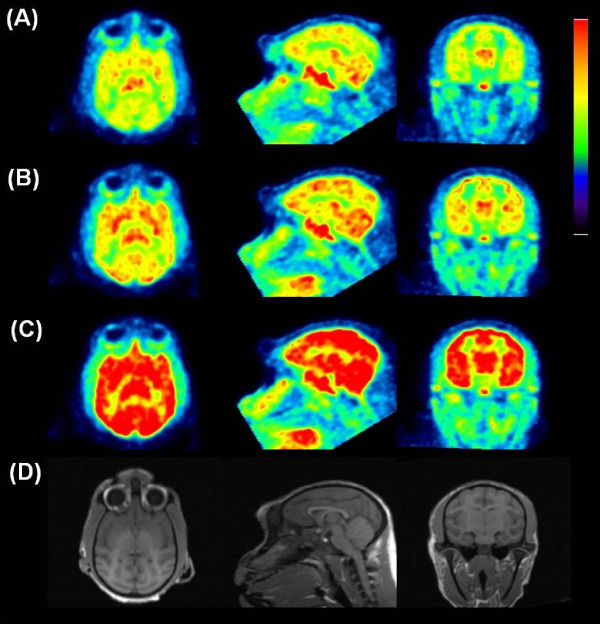
**PET images of a cynomolgus monkey. **PET images in the transaxial (left), the sagittal (middle), and the coronal (right) slices of the cynomolgus monkey, which were acquired from 9 to 123 min after intravenous injection with [^11^ C]*N*-methyl-SSR504734 under baseline (**A**), 0.5 mg/kg of pretreatment (**B**) and 1.5 mg/kg of pretreatment (**C**) conditions, and the corresponding MRI-T1 image (**D**).

**Figure 5:**
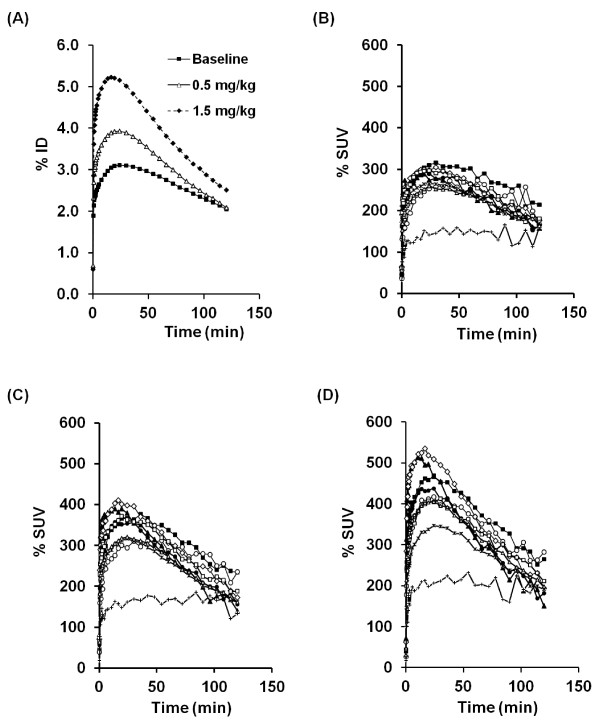
**Time-activity curves of brain radioactivity after intravenous injection of [**^**11**^ **C] *N* -methyl-SSR504734 in a cynomolgus monkey. **At baseline and pretreatment conditions (0.5 or 1.5 mg/kg of SSR504734). Whole brain uptake was expressed as percent injected dose (%ID) (**A**). Regional brain uptake was expressed as percent standard uptake value (%SUV) under baseline (**B**), pretreatment of SSR504734 (0.5 mg/kg) (**C**), and pretreatment of SSR504734 (1.5 mg/kg) conditions (**D**). Black square, thalamus; white circle, pons; black triangle, cerebellum; white diamond, putamen; asterisk, frontal cortex; cross, white matter; black circle, caudate; white square, anterior cingulate cortex; white triangle, temporal cortex.

The summation images are shown in Figure 4. In the baseline PET measurements (*N* = 2), time-activity curves (TACs) of ^11^ C]*N*-methyl-SSR504734 in whole brain reached peak (2.8%ID to 3.1%ID) at 24 min, followed by a moderate decline (Figure 5A). The rank order of regional brain uptake was as follows: thalamus > pons > cerebellum ≒ putamen > cortical regions > white matter (Figures 4A and 5B), which was basically consistent with *in vivo* regional brain uptake with other PET radioligands [19-21]. The radioactivity level of ^11^ C]*N*-methyl-SSR504734 was increased in all regions after pretreatment with SSR504734 in a dose-dependent manner (Figures 4B,C and 5C,D).

**Figure 6:**
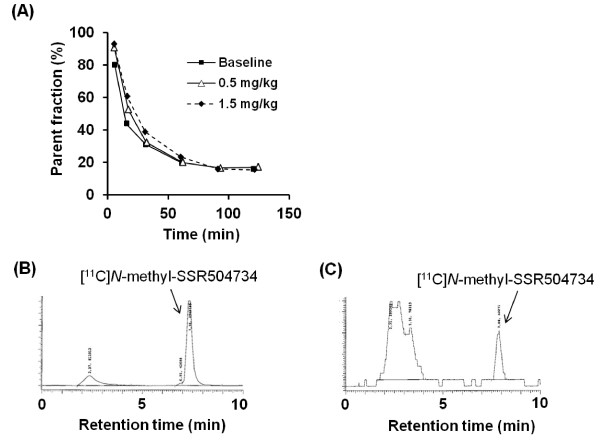
**Fraction of [**^**11**^ **C] *N* -methyl-SSR504734 in the plasma during PET measurements.** Percentage of unmetabolized [^11^ C]*N*-methyl-SSR504734 in the plasma of a cynomolgus monkey at baseline and pretreatment conditions (0.5 or 1.5 mg/kg of SSR504734) (**A**). HPLC analysis of plasma samples at 4 (**C**) and 90 min (**D**) after injection of [^11^ C]*N*-methyl-SSR504734 at baseline.

The parent fraction of [^11^ C]*N*-methyl-SSR504734 in the plasma was reduced during PET measurements. The metabolite rate was similar during baseline and pretreatment conditions with SSR504734 (Figure 6A). Radiochromatograms showed that more hydrophilic metabolites of [^11^ C]*N*-methyl-SSR504734 only were detected in the plasma (Figure 6B,C). Protein binding of [^11^ C]*N*-methyl-SSR504734 was 87% to 91% at baseline and after pretreatment.

### PET measurements using rhesus monkeys

The injected radioactivity was 170.0 ± 2.4 MBq. Specific radioactivity was higher than 353 GBq/μmol at the time of injection. The injected mass was less than 0.2 μg.

**Figure 7:**
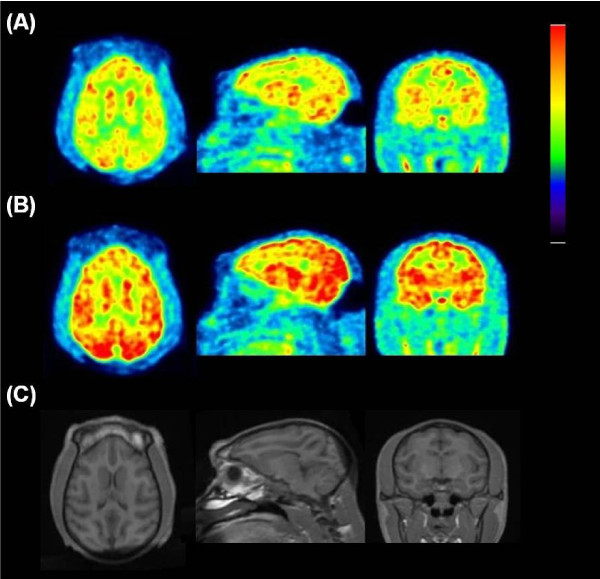
**PET images of rhesus monkey 2. **PET images in the transaxial (left), the sagittal (middle), and the coronal (right) slices of rhesus monkey 2 acquired from 9 to 123 min after intravenous injection with [^11^ C]*N*-methyl-SSR504734 at the baseline (**A**) and 4.5 mg/kg of pretreatment (**B** conditions, and the corresponding MRI-T1 image (**C**).

**Figure 8:**
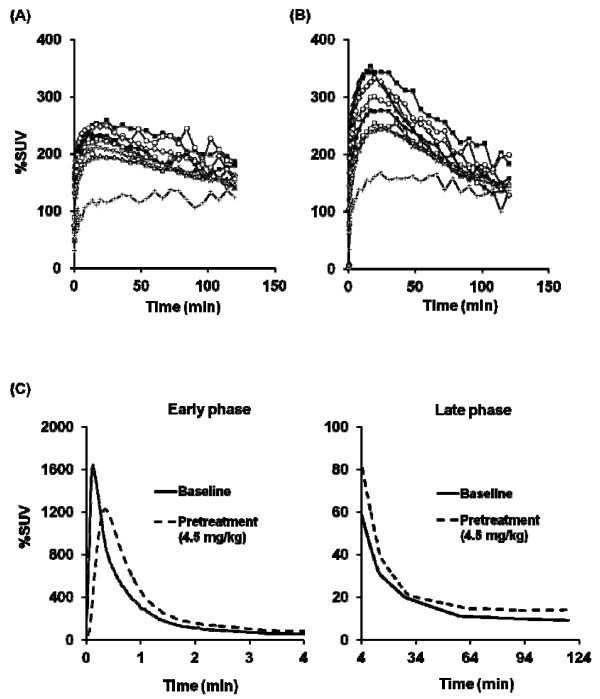
**Time-activity curves of brain radioactivity after intravenous injection of [**^**11**^ **C] *N* -methyl-SSR504734 in rhesus monkey 2. **(**A**) %SUV in brain regions at baseline condition. (**B**) %SUV in brain regions at pretreatment conditions (4.5 mg/kg of SSR5047). Black square, thalamus; white circle, pons; black triangle, cerebellum; white diamond, putamen; asterisk, frontal cortex; cross, white matter; black circle, caudate; white square, anterior cingulate cortex; white triangle, temporal cortex. (**C**) Time-activity curve (%SUV) of metabolite-corrected radioactivity in the plasma under baseline condition and pretreatment (4.5 mg/kg of SSR504734) condition.

In the baseline PET measurements (*N* = 2), TACs of [^11^ C]*N*-methyl-SSR504734 in the whole brain reached peak (3.2%ID, 172%SUV to 188%SUV) at 16.5 to 42 min. The rank order of regional brain uptake was as follows: thalamus > pons > putamen > frontal cortex≒temporal cortex > cerebellum> > white matter, as shown in Figures 7A and 8A. The brain distribution of [^11^ C]*N*-methyl-SSR504734 was similar to that of the cynomolgus monkey. Likewise, pretreatment with SSR504734 (1.5 and 4.5 mg/kg) caused increase of [^11^ C]*N*-methyl-SSR504734 uptake in the brain regions (Figures 7B and 8B) and considerable rise of the AUC of the arterial input function both at early and late phases (Figure 8C) in the PET studies of rhesus monkey 2. *V*_T_ values in the brain regions of the rhesus monkeys analyzed by 2TC were shown in Table 2. *V*_T_ could not be obtained robustly for the white matter. In the baseline PET measurement of rhesus monkey 1, *V*_T_ in GlyT1-rich regions such as the thalamus and pons were at a high level (31.0 to 35.2), whereas comparable *V*_T_ values were observed in other regions (23.1 to 28.6). After pretreatment of SSR504734 (1.5 and 4.5 mg/kg), *V*_T_ decreased approximately 20% and 33% reduction of *V*_T_ values in the whole brain as shown in Table 2.

**Table 2:** **Total distribution volume ( *V* **_**T**_**) in brain regions of the rhesus monkeys analyzed by 2TC model**

**Animal**	**Parameter**	**THA**	**Pons**	**CB**	**CAU**	**PUT**	**FC**	**ACC**	**TC**	**WB**
Rhesus monkey 1	*V*_T _(baseline)	31.0	35.2	23.7	26.2	26.7	25.2	28.6	25.6	23.1
	*V*_T _(Blocking (1.5 mg/kg))	29.6	26.8	20.4	22.0	22.8	20.6	22.5	21.8	18.6
	% decrease of *V*_T_^a^	4.5	23.9	13.9	16.0	14.6	18.3	21.3	14.8	19.5
Rhesus monkey 2	*V*_T _(baseline)	15.7	16.9	12.8	17.0	15.8	14.3	15.5	12.3	12.6
	*V*_T _(Blocking (4.5 mg/kg))	11.9	10.9	9.0	9.2	10.1	8.6	9.0	8.9	8.5
	% decrease of *V*_T_^a^	24.2	35.5	29.7	45.9	36.1	39.9	41.9	27.6	32.5

^a^Percent decrease of values from baseline study to blocking study. *ACC*, anterior cingulate cortex; *CAU*, caudate; *CB*, cerebellum; *FC*, frontal cortex; *PUT*, putamen; *TC*, temporal cortex; *THA*, thalamus; *WB*, whole brain.

**Figure 9:**
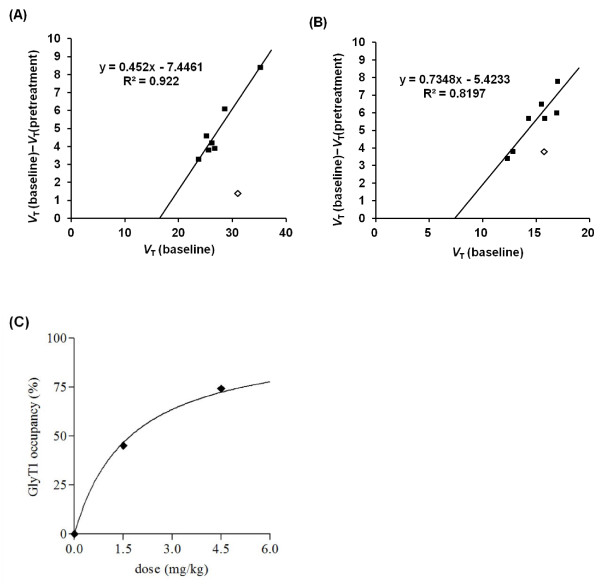
**Lassen plot analysis applied to PET occupancy studies. **Data were taken from [11 C]N-methyl-SSR504734 studies in the rhesus monkey brain at baseline and after administration of SSR504734 at doses of 1.5 mg/kg in rhesus monkey 1 (**A**) and 4.5 mg/kg in rhesus monkey 2 (**B**). Thalamus (white diamond) was not included for Lassen plot. (**C**) Relationship between GlyT1 occupancy (in percent) and dose of SSR504734. The relationship between GlyT1 occupancy (in percent) and dose of SSR504734 was expressed by the equation: $\text{Occupancy }\left(\%\right)=O_{\text{max}}\times \frac{C\text{p}}{C\text{p}+K\text{d}}$, where Omax is the assumed maximal occupancy (i.e., 100%), and Cp is the dose at which occupancy becomes 50%. Kd was estimated to be 1.7 mg/kg.

Using Lassen plot, a linear fitting to the data points from seven brain regions was obtained for the dose of 1.5 and 4.5 mg/kg of SSR504734 (Figure 9A,B). Target occupancy was calculated to be 45.2% and 74.3% at 1.5 and 4.5 mg of SSR504734, respectively (Figure 9C).

The estimated *V*_ND_ values from the graphical analysis in Figure 9A,B were 16.5 and 7.5, respectively. Percentage of *V*_ND_ in *V*_T_ was 52% to 66% and 39% to 56% at 1.5- and 4.5-mg doses of SSR504734, respectively (Table 3).

**Table 3:** **Percentage of *V* **_**ND **_**in total distribution volume in rhesus monkeys**

**Study**	**Parameter**	**Pons**	**CB**	**CAU**	**PUT**	**FC**	**ACC**	**TC**
Baseline + blocking (1.5 mg/kg)	*V*_T_	31	35.2	23.7	26.2	26.7	25.2	28.6
	*V*_ND_	16.5	16.5	16.5	16.5	16.5	16.5	16.5
	% of *V*_ND _in *V*_T_	66	50	55	55	52	59	54
Baseline + blocking (4.5 mg/kg)	*V*_T_	16.9	12.8	17	15.8	14.3	15.5	12.3
	*V*_ND_	7.5	7.5	7.5	7.5	7.5	7.5	7.5
	% of *V*_ND _in *V*_T_	56	42	56	53	48	52	39

*ACC*, anterior cingulate cortex; *CAU*, caudate; *CB*, cerebellum; *FC*, frontal cortex; *PUT*, putamen; *TC*, temporal cortex; *V*_ND_, nondisplaceable distribution; *V*_T_, volume distribution volume.

## Discussion

In the present study, ^11^ C]*N*-methyl-SSR504734 was demonstrated to be a promising PET radioligand to measure GlyT1 occupancy in nonhuman primates. ^11^ C]*N*-methyl-SSR504734 was synthesized successfully using ^11^ C]CH_3_OTf with moderate radiochemical yield and ultrahigh specific radioactivity compared with previously reported GlyT1 imaging [19,20,23].

In the *in vitro* ARG studies of human brain slices, ^11^ C]*N*-methyl-SSR504734 showed specific binding in GlyT1-rich regions such as the white matter and pons. However, as only small decrease of ^11^ C]*N*-methyl-SSR504734 binding was detected, nonspecific binding was expected to be high in all brain regions (Figure 3). Previously, ^125^I]IMPB, which is a ^11^ C]*N*-methyl-SSR504734 analog, showed excellent high specific binding to rat brain slices under *in vitro* ARG studies. In addition, both IMPB and *N*-methyl-SSR504734 showed very high inhibitory activity against the human GlyT1 (IMPB, IC_50_ = 2.4 nM; *N*-methyl-SSR504734, IC_50_ = 7.5 nM) as determined in a ^3^ H]glycine uptake assay using JAR cells [24]. Therefore, it was suggested that this series of compounds can bind to both rodent and human GlyT1. The present human ARG results seemed not to be in line with the previous report [24]. Several factors such as species differences and condition of the ARG slices should be taken into consideration.

In PET studies using cynomolgus and rhesus monkeys, ^11^ C]*N*-methyl-SSR504734 showed high brain uptake and a regional brain distribution consistent with other GlyT1 PET ligands [19-21]. The low ^11^ C radioactivity in the white matter, where GlyT1 binding was reported to be high in *in vitro* ARG studies, was consistent with our previous *ex vivo* studies of ^125^I]IMPB [24] and previous PET studies with other GlyT1 ligands [19-21]. Compared with *in vitro* ARG results, low uptake in the white matter might be related to low blood flow in the white matter [33,34].

*V*_T_ values of white matter could not be obtained reliably with the 2TC model. Although several other quantitative methods such as Logan plot were investigated, they did not work well (data not shown). This unreliable estimation of *V*_T_ might be related in part to time-activity curves in the white matter being relatively noisy due to potential spillover from the cortex and, in part, to [^11^ C]*N*-methyl-SSR504734 kinetics being slower in the white matter than in other brain regions.

Although over 70% of [^11^ C]*N*-methyl-SSR504734 was metabolized within 90 min after injection of [^11^ C]*N*-methyl-SSR504734, only higher hydrophilic fraction was observed (Figure 6B,C). Because these metabolites of [^11^ C]*N*-methyl-SSR504734 are unlikely to penetrate the blood brain barrier, they are expected to have little influence on the *in vivo* brain distribution pattern of [^11^ C]*N*-methyl-SSR504734.

The radioactivity concentration in the brain increased after pretreatment with SSR504734 in both the cynomolgus and rhesus monkeys. ^11^ C]SA1, which is a structurally analogous compound of ^11^ C]*N*-methyl-SSR504734, has been reported to show similar increase in brain uptake after pretreatment with SSR504734 in rhesus monkeys [23]. ^11^ C]SA1 did not show the conclusive decrease of *V*_T_ due to large variability of *V*_T_. On the other hand, distribution volumes of ^11^ C]*N*-methyl-SSR504734 were found to decrease after pretreatment with SSR504734 in a dose-dependent manner. In the monkeys, ^11^ C]*N*-methyl-SSR504734 showed faster receptor kinetics than ^11^ C]SA1, thereby providing the equilibrium between brain and plasma, and allowing more reliable estimation of *V*_T_ values.

In the Lassen plot analysis, seven regions were used for occupancy calculation. The thalamus seemed to behave differently from other regions in the plot, indicating that the thalamus might have a different nondisplaceable distribution volume or a different GlyT1 occupancy.

The estimated target occupancy by SSR504734 was increased dose-dependently (Figure 9), indicating that [^11^ C]*N*-methyl-SSR504734 could be a promising PET ligand for occupancy studies of GlyT1 blockers.

^11^ C]GSK931145 demonstrated good brain penetration and usefulness for GlyT1 occupancy study in monkeys [21]. However, poor test-retest results were reported in human subjects. It might be related to a little difference in the brain kinetics of ^11^ C]GSK931145 between monkey and human subjects. It would be important to investigate whether there is species difference in the brain kinetics of ^11^ C]*N*-methyl-SSR504734 between monkey and human subjects.

The present monkey PET study was performed under anesthesia induced by ketamine and maintenaned by sevoflurane. As ketamine is known to block NMDA receptors as an open channel blocker, and sevoflurane is a noncompetitive antagonist of NMDA receptor, an indirect interaction between these two anesthesia drugs and glycine transporter 1 function via NMDA receptors cannot be excluded. The use of anethesia should be taken into account when the radioligand is considered for further evaluation.

In the present study, SSR504734 was used as a blocker. As SSR504734 is a structurally analogous compound to [^11^ C]*N*-methyl-SSR504734, the possibility of interaction at a nonspecific binding site was not completely excluded. If the blocking occurs at a nonspecific binding site, estimated *V*_ND_ by Lassen plot would be close to zero. Considering the estimated *V*_ND_ by Lassen plot shown in Figure 9, the possibility were considered to be low. To exclude the possibility completely, blocking study by compounds with different chemical structure should be performed in future study.

## Conclusions

In this study, [^11^ C]*N*-methyl-SSR504734 was successfully synthesized and evaluated as a candidate PET radioligand for GlyT1. [^11^ C]*N*-methyl-SSR504734 showed a regional accumulation, which was consistent with previous reports on the GlyT1 distribution. *V*_T_ values were decreased by SSR504734 in a dose-dependent manner, suggesting specific binding. Considering these results, [^11^ C]*N*-methyl-SSR504734 is a promising PET radioligand for GlyT1. The present study warrants further PET studies in human subjects.

## Competing interests

The authors declare that they have no competing interests.

## Authors’ contributions

TF, AT, and CH carried out the design of this study and drafted the manuscript. TF, YM, MH, and MN participated in the synthesis of SSR504734 and *N*-methyl-SSR504734. TF, ZJ, and JDA carried out the radiosynthesis of [^11^ C]*N*-methyl-SSR504734. TF and BG carried out the autoradiography studies. AT, BG, SJF, and JDA participated in the PET studies. RN carried out the metabolite analysis and measurement of protein binding. AT and CH participated in the data analysis, data interpretation, and revising of the manuscript. All authors read and approved the final manuscript.
